# The IMPROVE trial: study protocol for a pragmatic cluster randomised controlled trial to assess the effectiveness of using lay health workers to improve uptake and completion of pulmonary rehabilitation in patients with chronic obstructive pulmonary disease

**DOI:** 10.1186/s13063-024-07998-x

**Published:** 2024-03-19

**Authors:** Gill Gilworth, Katherine Harris, Toby L. Morgan, Salma Ayis, Julia Fox-Rushby, Emma Godfrey, Nicholas S. Hopkinson, Simon Lewin, Ka Keat Lim, Arietta Spinou, Stephanie J. C. Taylor, Patrick White

**Affiliations:** 1 Academic Department of Rehabilitation Medicine, Faculty of Medicine and Health, University of Leeds, Sheffield, UK; 2 School of Life Course & Population Sciences, Faculty of Life Sciences & Medicine, King’s College London, Addison House, London, SE1 IUL UK; 3 School of Mental Health and Psychological Sciences, Institute of Psychiatry, Psychology & Neuroscience, Faculty of Life Sciences & Medicine, King’s College London, London, UK; 4 National Heart and Lung Institute, Imperial College London, London, UK; 5 Department of Health Sciences Ålesund, Faculty of Medicine and Health Sciences, Norwegian University of Science and Technology (NTNU), Trondheim, Norway; 6 School of Life Course & Population Sciences, Faculty of Life Sciences & Medicine, King’s Centre for Lung Health, King’s College London, London, UK; 7 Wolfson Institute of Population Health, Barts and The London School of Medicine and Dentistry, Queen Mary University of London, London, UK; 8 Health Systems Research Unit, South African Medical Research Council, Cape Town, South Africa; 9 Centre for Epidemic Interventions Research, Norwegian Institute of Public Health, Oslo, Norway

**Keywords:** Cluster randomised controlled trial, Lay health workers, Pulmonary rehabilitation, Chronic obstructive pulmonary disease

## Abstract

**Background:**

Pulmonary rehabilitation (PR) is a programme of exercise and education and the most effective treatment for the symptoms and disability associated with chronic obstructive pulmonary disease. However, the benefits of PR are limited by poor uptake and completion. This trial will determine whether using trained volunteer lay health workers, called “PR buddies,” improves uptake and completion of PR and is cost-effective. This trial protocol outlines the methods for evaluating effectiveness, cost-effectiveness, and acceptability.

**Methods:**

The IMPROVE trial is a pragmatic, open, cluster randomised controlled trial planned in 38 PR services across England and Wales. PR services will be randomised to either intervention arm—offering support from PR buddies to patients with chronic obstructive pulmonary disease—or to usual care as the control arm. PR staff in trial sites randomised to the intervention arm will receive training in recruiting and training PR buddies. They will deliver training to volunteers, recruited from among people who have recently completed PR in their service. The 3-day PR-buddy training programme covers communication skills, confidentiality, boundaries of the PR-buddy role and behaviour change techniques to help patients overcome obstacles to attending PR. An internal pilot will test the implementation of the trial in eight sites (four intervention sites and four in control arm). The primary outcome of the trial is the uptake and completion of PR. A process evaluation will investigate the acceptability of the intervention to patients, PR staff and the volunteer PR buddies, and intervention fidelity. We will also conduct a cost-effectiveness analysis.

**Discussion:**

Improving outcomes for chronic obstructive pulmonary disease and access to PR are priorities for the UK National Health Service (NHS) in its long-term plan. The trial hypothesis is that volunteer PR buddies, who are recruited and trained by local PR teams, are an effective and cost-effective way to improve the uptake and completion rates of PR. The trial is pragmatic, since it will test whether the intervention can be incorporated into NHS PR services. Information obtained in this trial may be used to influence policy on the use of PR buddies in PR and other similar services in the NHS.

**Trial registration:**

ISRCTN12658458. Registered on 23/01/2023.

**Supplementary Information:**

The online version contains supplementary material available at 10.1186/s13063-024-07998-x.

## Introduction

### Background and rationale

Chronic obstructive pulmonary disease (COPD) is the most common chronic lung disease caused by smoking [1]. More than a million people in the United Kingdom (UK) live with the condition, with a higher prevalence typically reported in more deprived areas [2, 3]. The symptoms of COPD include cough, breathlessness and fatigue [4]. A severe exacerbation of COPD symptoms is a key cause of attendances at Accident and Emergency departments and of hospital admissions in the UK [5]. COPD is also the third most common cause of death in England and Wales [6].

The most effective treatment for the symptoms and disability associated with COPD is pulmonary rehabilitation (PR) [7]. PR is a prescribed exercise-based treatment programme which has been identified by NICE (The National Institute for Health and Care Excellence) as one of the “five fundamentals of COPD care” [8, 9]. Structured group classes include physical exercise and information on self-management. Social interaction is a notable element in almost all services. The effectiveness of PR in improving symptoms, quality of life and exercise capacity in people living with COPD has been demonstrated conclusively in a longstanding Cochrane review now close [10]. More recently, PR has also been associated with improved survival following discharge from hospital after a COPD exacerbation [11].

Despite strong evidence demonstrating the effectiveness of PR, patients likely to benefit from it often do not receive a referral by healthcare professionals or do not join the programme. In an audit of PR services in England and Wales in 2015, the annual number of referrals of patients with COPD to 240 services was estimated to be 68,000 [12]. This equated to approximately 5% of the estimated 1.3 million people with symptomatic COPD. If patients were referred every 5 years (annual referral is desirable), the rate of referral of appropriate people with COPD would be still only 25%. In subsequent audits, 62% of the eligible patients who were referred to PR joined the programme, and 65% of those who joined the programme completed PR, an overall rate of uptake and completion of 40% [12]. The American Thoracic Society and European Respiratory Society have recommended a more collaborative approach between clinicians, patients, and funders to improve the delivery of PR within healthcare services [13].

The barriers to attending or completing PR are well understood. The reasons for poor rates of uptake include a perceived lack of benefit by patients and uncertainty about its effectiveness by those making the referral, travel issues, inconvenient timing and disruption to valued routines [14]. Reasons such as being a current smoker and having comorbidities, particularly depression, are associated with non-completion [15]. A qualitative study of patients who had recently completed PR reported that smoking was associated with feelings of unworthiness to participate in the programme [16].

Previous interventions that have sought to improve uptake and completion of PR have included computerised reminders, provision of a patient information or care manual, education for clinicians and financial incentives [17]. In 2009, Harris et al. showed in a before and after study that providing patients with a manual summarising evidence of effective care led to an 18% improvement in PR enrolment in the most socio-economically disadvantage group, compared to usual care [18]. Zwar et al. showed increased uptake of PR among patients with COPD who received care for their COPD at home by a nurse (31.1% compared to 9.6% for the control group) [19].

Lay health workers are described as “any health worker carrying out functions related to healthcare delivery, trained in some way in the context of the intervention, and having no formal professional or paraprofessional certificate or tertiary education degree” and can be involved in either paid or voluntary care [20]. Lay health workers have been used predominantly in low- and middle-income countries where their role has been to complement local health services especially where there is underfunding of conventional health services. There is evidence that using lay health workers improves uptake of clinical [21], but they are not widely used in the NHS. In the United States of America, lay health workers commonly work as patient navigators and play a range of other roles such as supporting patient self-management [22]. Adhikari et al. have published the protocol for a cluster randomised controlled trial of female volunteers in the diagnosis and management of COPD in Nepal [23]. In the context of the trial proposed here in the UK, lay health workers are an example of reverse innovation, introducing into high-income countries an innovation that was first devised in low- and middle-income countries [24].

The IMPROVE trial seeks to use lay health workers to support patients with COPD who are referred for PR to help them overcome barriers that could stop them from attending the programme. In this intervention, we train the lay health workers to use selected behaviour change techniques to overcome these barriers. In the trial, lay health workers are called “PR buddies,” a term preferred by our patient advisors. In a feasibility study of using lay health workers to improve the uptake and completion of PR, the feasibility of recruitment and training of PR-buddy volunteers was demonstrated [25]. PR buddies were successfully used in supporting patients and the fidelity of the intervention was shown [26]. Among the advantages of using PR buddies are the shared social backgrounds, and shared personal experiences of the health issue among patients and PR buddies [27].

### Objectives

The primary objective of the IMPROVE trial is to assess the effectiveness of the intervention using volunteer PR buddies trained by PR teams to improve completion of PR in patients with COPD. The working hypothesis for the trial is that PR experienced volunteer PR buddies, recruited and trained by PR staff to support COPD patients newly referred to PR, will improve uptake and completion of PR in comparison with usual care.

The secondary objectives include:

1. To assess the intervention fidelity with respect to:
  - The training of PR teams to set up and run a PR-buddy service
  - The recruitment and training of PR buddies by the PR teams
  - The delivery of the support intervention to patients by the PR buddies
2. To assess the acceptability of the intervention to trial participants, including PR staff, PR buddies and patient participants.
3. To identify PR service, PR-buddy and patient-participant factors associated with the implementation, delivery and impact of the intervention.
4. To conduct within-trial and longer-term cost-effectiveness analyses of the intervention compared to standard care.

The trial includes an internal pilot being conducted in eight sites, four in the intervention arm and four in the control arm. The aims of the pilot are:

- Testing of the PR staff training (intervention sites)
- Testing the feasibility of PR teams recruiting and training a group of PR buddies (intervention sites)
- Implementation of a PR-buddy support service to COPD patients referred for PR (intervention sites)
- Recruitment of patient participants (all sites)

### Trial design

IMPROVE is a prospective, open, pragmatic, cluster randomised controlled clinical trial, with an internal pilot, with clustering at site level. Sites will be PR services. The feasibility study suggested that the recruitment and training of PR buddies and the recruitment of patient participants are feasible [26]. Nonetheless, the trial is in equipoise about the effectiveness of the intervention. Trial sites will be randomised 1:1 to either PR-buddy service or to continue delivering care as usual.

## Methods: participants, interventions and outcomes

### Study setting

The IMPROVE trial will be conducted in PR centres in England and Wales. It will recruit PR services (sites) being run either by NHS staff, or by private companies contracted by the NHS. These may be community or hospital-based services. Potential sites are being identified through the list of sites participating in the National Respiratory Audit Programme and through the Clinical Research Network and regional PR networks. Each site is then contacted by a member of the IMPROVE research team. There will be an internal pilot in eight sites; the target is for the main trial to have a further 30 sites making 38 sites in total. The list of current participating sites can be obtained from the trial website: https://improvetrial.co.uk/get-involved.

### Eligibility criteria

#### Inclusion criteria

There are four groups of participants in the IMPROVE trial:

(A) Trial sites
(B) Individual PR staff at sites randomised to the intervention arm
(C) Volunteer PR buddies (intervention sites)
(D) Patient participants (all sites)

The intervention includes elements that test the efficacy of the central component of the intervention: the support of referred patients by PR buddies. It also includes elements that test the capacity for the intervention to be implemented in PR services. Because the PR staff who receive trial-specific training will deliver the training elements of the intervention to PR buddies, they will also be trial participants.

Category A—Trial sites (PR services)

1. Provide > 15 pre-PR assessment* appointments per month (to allow for recruitment of sufficient patient participants)
2. Collect baseline and final PR assessment data for participating patients
3. Have a PR completion rate ≤ 55% determined by count based on appropriate referrals received, or completion rate ≤ 65% determined by count based on patients who attend pre-PR assessment
4. Agree to be randomised to intervention or usual care
5. Include eligible patients in the invitation to be randomised for the PR-buddy intervention or usual care (researcher taking consent to be blinded to outcome of randomisation for the site)
6. Intervention sites agree to release at least three PR staff for training; at least two of three participating staff members to be a registered healthcare professional or educated to degree level in an appropriate subject
7. One or more members of the PR team to have at least 1 year’s experience in PR

*A pre-PR assessment is carried out by PR services for each referred patient to assess their suitability and record baseline data including exercise capacity. This assessment enables the exercise element of PR to be tailored to the individual.

Category B—Individual PR staff at sites randomised to the intervention arm

1. Aged 18 years or above
2. Employed members of staff within the PR service at the intervention site
3. Willing to undertake 2½ days of training and to train, recruit, manage and support PR buddies
4. Willing to recruit, train and then manage and support PR buddies over a nine-month period
5. Willing to take part in research activities including keeping accurate records

Category C—Intervention site PR-buddy volunteers

1. Aged 18 years or above
2. Have a diagnosis of COPD and have completed PR within the previous year
3. Volunteer for the role
4. Undertake training and be supervised by the PR staff
5. Willing to support at least 6 PR patients over up to 9 months
6. Able to travel independently
7. Willing to use encrypted smart phones (after training) for recording conversations with supported patient participants (to enable evaluation of the intervention fidelity)

Category D—Patient participants

1. Aged 18 years or above
2. Have a diagnosis of COPD
3. Be referred to the PR service of one of the participating sites
4. Have a modified Medical Research Council (mMRC) breathlessness score > 1
5. Consent to be randomised to intervention or usual care arm of the trial
6. Consent to receive telephone contact by PR buddy and to meet when appropriate (intervention arm sites)
7. Consent to provide personal details to the IMPROVE research team
8. Consent to provide information on PR attendance to the IMPROVE research team, and to answer questionnaires on personal characteristics, impacts of PR on their health, health economic data, and data on their use of NHS services

#### Exclusion criteria

Category A—Trial sites (PR services)

1. Unable to identify at least three members of staff who are willing to consent to participation in the trial
2. Local NHS health authority Research and Development (R&D) office unwilling to support the trial

Category B—Intervention site participating PR staff

Not expected to be employed in their post for the duration of PR staff training and PR-buddy training at the site.

Category C—Intervention sites PR-buddy volunteers

1. Unable to participate for the duration of the trial at their site
2. Unable to travel independently to meet patient participants
3. Unable or unwilling to use a smart mobile phone
4. Unable to give valid informed consent
5. Failed Disclosure and Barring Service (DBS) check
6. Pregnant or breastfeeding
7. Not deemed suitable to work with patient participants, as assessed by a Health Care Professional

Category D—Patient participants

1. Have poorly controlled angina on minimal exertion (defined as history of heart pain when walking on level ground)
2. Have had a myocardial infarction in the 6 weeks prior to being approached to give consent
3. Have breathlessness as a result of cardiac disease
4. Have uncontrolled hypertension
5. Have any medical problem that severely restricts exercise or compliance with the programme
6. Unable to give valid informed consent
7. Pregnant or breastfeeding

### Consenting participants

PR-staff research participants will be in the intervention arm of the trial. They will give written informed consent after the site has been randomised. Sufficient time will be allowed for the invited staff to read the participant information sheet and to have the opportunity to ask questions. Consent will be taken by a member of the IMPROVE research team on a video call. Volunteers who would like to become PR buddies will give written informed consent in-person during the first day of the PR buddies’ training having previously been sent the participant information sheet. Consent will be taken by a trained member of PR staff or a member of the IMPROVE research team after the PR buddy has had a chance to ask questions.

Patient participants will give their consent to local National Health Service (NHS) research delivery staff or Clinical Research Network (CRN) staff. Patient participant consent may be taken in-person or remotely through a phone or video call, depending on individual preference. All potential participants will be sent the patient information sheet and the consent form in advance of consent meetings. After having an opportunity to ask questions, if the patient consents they will sign and date the consent form. In in-person consent interviews, the research staff member will countersign and date the form and give a copy to the patient. Where consent is given remotely, two methods may be used. The patient may sign and date the consent form and send it to the research staff member who will then sign and date it and send a copy to the patient. The alternative method is for the research staff member to audio-record the patient giving verbal consent by telephone and to securely store the recording. The research staff member should sign the consent form and send a copy to the patient. [Communication from the Preston Research Ethics Committee, 19 April 2023].

As part of the trial process evaluation, some patient participants and some PR staff will be invited to a qualitative interview, and some PR buddies will be invited to participate in focus groups to provide feedback on their experience of participating in the trial. Participants consenting to give one-to-one interviews or to participate in a focus group will additionally consent to these interactions being recorded and transcribed. Consent to participate in a qualitative interview or focus group will be taken at the same time as written informed consent for participation in the trial as described above.

This trial does not involve the use of any biological specimens.

## Intervention

Randomisation will be at the PR-service level. Rates for completion of PR for patient participants who receive PR-buddy support in services in the intervention arm will be compared to those for patient participants in PR services randomised to offer care as usual. Randomisation at the level of sites is essential to prevent contamination of patient participants not receiving the PR-buddy intervention by those who are receiving it or who have received it. For this reason, the comparison will be between sites and not between individual patient participants. Recruitment of patient participants will be identical in both arms of the trial. Patient participants will not be aware of the outcome of the PR service randomisation at the time of recruitment. The site target for recruitment of patient participants, at least 36, will be the same for all sites.

The IMPROVE PR-buddy intervention has five elements: PR-staff training, PR-buddy recruitment, PR-buddy training, patient-participant support by the PR buddies, and PR-buddy management and support by the PR staff. A ‘train-the trainer’ approach will be used, whereby designated PR staff in services in the intervention arm of the trial will be trained in the setting up and running of a PR-buddy service. Using this model moves the burden of training multiple groups of PR buddies from the IMPROVE research team to individual PR services. This will help to provide a more realistic assessment of the intervention as it would be implemented in ‘real-world’ practice and will also facilitate implementation across the NHS if the trial outcome is positive.

The PR-buddy training will be informed by the COM-B model and include selected behaviour change techniques. The COM-B model for behaviour change proposes that there are 3 components to any behaviour: capability (C), opportunity (O), and motivation (M) [28–30]. PR buddies sharing their own positive experiences of PR with patient participants will be a central element of the PR-buddy intervention. Manuals for the training for PR staff and the training of PR buddies will be freely available for services in the NHS only after completion of the trial.

The intervention includes the following steps:

- *Step 1*: Training will be delivered to at least three PR staff members from each intervention site by the IMPROVE team. The 2.5 days PR staff training comprises a half day self-directed learning session, 1 day “remote live”; by which we mean that the participants will attend the training at the same time, but it will be delivered remotely using Microsoft Teams. The final day will be in-person training.
- *Step 2*: PR staff will recruit volunteer PR buddies by writing to people with COPD who completed PR in their service in the last year. Patients completing their end of PR assessment will also be informed about the IMPROVE trial. PR staff will screen interested volunteers by phone. Selected volunteers will be asked to complete an application form and will be invited for an in-person interview by PR staff. At interview, the suitability of volunteers for the role will be checked. Candidates will either be offered a place on the PR-buddy training course or signposted to other volunteering opportunities.
- *Step 3*: PR staff will train the PR buddies in supporting patients who are referred for PR. The focus will be on training PR buddies to identify and work with patients to resolve potential barriers to attending PR including the PR buddies sharing their own positive experiences of PR with patient participants. Communication skills, confidentiality and boundaries of the role will also be included. The training will be delivered in-person once a week for 3 weeks.
- *Step 4*: PR staff will allocate PR buddies to patient participants, and the PR buddies will make initial contact by phone. PR buddies and supported patients will be encouraged to also meet in person if both agree. Mean number of encounters (telephone and face to face) is expected to be about six [25].
- *Step 5*: PR buddies will have monthly group support meetings facilitated by the trained PR staff. At these meetings, the PR buddies will have opportunities to share experiences, recap elements of the PR-buddy training and allow PR staff to ensure that the PR buddies’ workload is acceptable and that the contact they are having with their patient participants is sufficient.

### Criteria for discontinuing or modifying the interventions

If a PR staff member is unable to attend any part of their training, an alternative session will be provided, if possible, at another training session. If a staff member is unable to continue in the trial, another PR staff member who has attended training will take over the role. If a PR buddy is unable to attend any part of the training, the component(s) missed will be noted and, where possible, catch-up sessions will be arranged. If missed training sessions cannot be caught up, the volunteer(s) will be signposted to other volunteering opportunities.

Following training, if a PR buddy is unable to continue supporting patients the patients will be allocated a different PR buddy. If a PR buddy asks to be withdrawn from support of a particular patient participant, or a patient participant asks to be withdrawn from receiving support from a particular PR buddy, the party seeking to be withdrawn will be interviewed by a PR staff member to assess the issue. If there are no concerns about the behaviour of the other party, then the staff member will recommend that new PR-buddy support or a new patient participant will be found to replace the withdrawing party.

### Strategies to improve adherence to interventions

Attendance registers will be kept for both PR-staff and PR-buddy training. Checklists will be used to record the required elements of the training delivered. Each remote live day of PR-staff training and at least 1 session (1/4 of a day) of the PR-buddy training at each site will be recorded by the IMPROVE research team to enable more objective assessment of fidelity of delivery of training. A 20% sample will be analysed for content of delivery by listening back to of these recordings and comparing them to the relevant component of the training manual and slide deck. Training reviews will be used both for PR staff and PR buddies to assess fidelity of receipt of the training.

Using dedicated trial phones, PR buddies will record the contacts they have with the patients they support. Recordings will be collected once a month by the research team. Intervention fidelity of PR-buddy support will be assessed by listening to a randomly selected sample of the recordings (10% of the recordings made by 2 PR buddies per site). This assessment will focus on analysing delivery style (use of communications skills training) and on the use of taught behaviour change techniques by the PR buddies. Analysis will be based on a coding framework developed and used in the feasibility study [25]. Recordings will be coded independently by two coders and discrepancies in coding then resolved by discussion.

Any concomitant care is permitted. It is not anticipated that provisions will be required for post-trial care. This trial has been assessed as low risk. The trial co-sponsors have agreed that any harm resulting from the trial will be the responsibility of the main sponsor, King’s College London. King’s College London will provide indemnity for the duration of the trial.

### Outcomes

The primary outcome is completion of PR by each individual patient participant, summarised as a proportion for each trial site. Standard PR treatment usually consists of a pre-PR assessment, twelve classes over 6 to 8 weeks and a final assessment. Uptake is defined as attendance at the pre-PR assessment; completion is defined as attendance of 75% or more of the planned PR sessions—usually at least 9 out of 12 sessions [31, 32].

The secondary outcomes are:

1. The proportions of PR sites that were recruited and that were retained—measured through a site recruitment log and a record of withdrawal/default.
2. Demographic and clinical characteristics of PR buddies in participating sites measured through data collected and recorded on the PR-buddy application form.
3. The proportions of patient participants that were recruited and retained—measured through a site log rate of acceptance of the invitation to participate, rate of assessment for PR and rate of participants followed through to completion.
4. Demographic and clinical characteristics of patient participants in participating sites measured through data collected and recorded on Case Report Forms (CRFs) at recruitment and consent interview.
5. Exercise capacity being assessed at the pre-PR assessment, respiratory-specific quality of life and psychological wellbeing measured at baseline, at 3 months and at 6 months in a questionnaire. Exercise capacity will be measured through site records of the 6-min walking distance test or the incremental shuttle walk test or the 60-s sit to stand test [33–35]. Quality of life and psychological wellbeing will be measured by the Chronic Obstructive Pulmonary Disease Assessment Test and the Hospital Anxiety and Depression Scale in a postal questionnaire [36, 37]. Breathlessness will be measured by the Medical Research Council breathlessness questionnaire [38].

The process evaluation will include collection of process data (e.g. relating to training delivery, contacts between PR buddies and supported patients), qualitative interviews with some of the trial participants (PR staff and patient participants), focus groups with some PR buddies and field diaries to capture reflections of researchers throughout the implementation period. Assessment of the fidelity of the intervention delivery of patient support by PR buddies is as described above. We will also perform an economic evaluation to examine whether the intervention is potentially cost-effective compared to the current standard care alone, from the perspectives of the NHS and personal social services and the society.

### Participant timeline

**Fig. 1:**
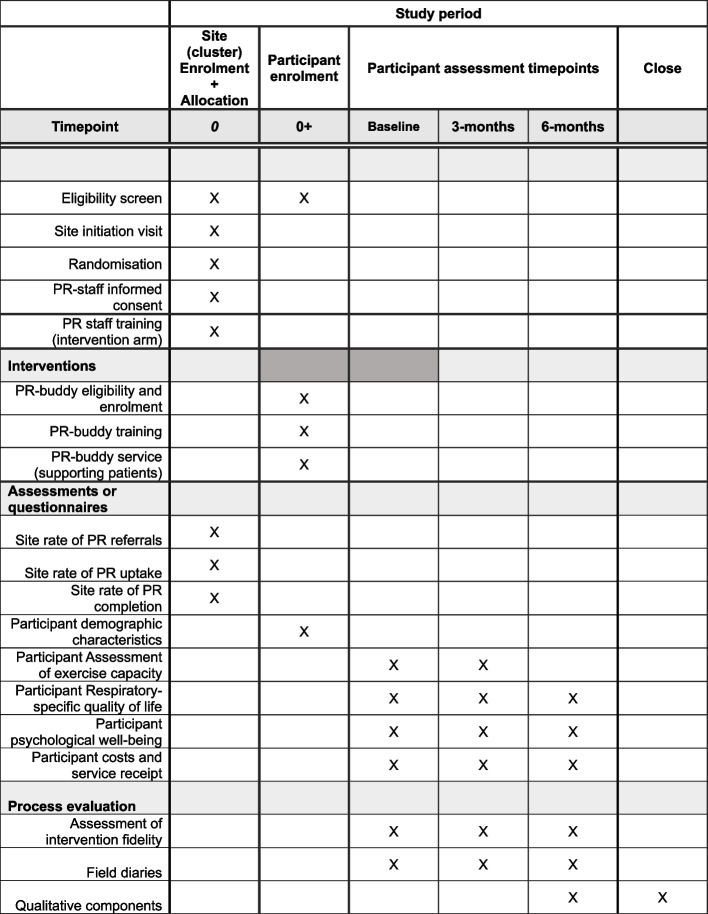
Timeline of participation in the study (Spirit Figure)

Figure 1 (Spirit Figure) shows the timeline for enrolment and participation in the study. PR sites, PR buddies and patient participants will be enrolled for approximately 9 months.

### Sample size

Based on the 2017 PR audit of the Royal College of Physicians the mean completion proportion over 182 PR sites in England was 40% [12]. Sites with a completion proportion of 55% or less were considered “sub-optimal performers”; the mean completion proportion among these sites was estimated at 32%. Due to regression to the mean, it is likely that these “sub-optimal” performing sites may perform better the following year, and so the control group completion proportion is likely to be between 32 and 40%.

Assuming an overall completion proportion of 40% in the control group, 36 sites (18 per arm) would be required to detect a 40% relative increase in completion proportion to 56% with 90% power, assuming an intraclass correlation coefficient (ICC) of 0.056 and 30 patient participants per site. The choice of ICC = 0.056 was made on basis of the literature and recommendations based on similar interventions [39]. Where interventions include training of health professionals, or other personnel who will deliver the intervention while the outcome is to be measured on patients, an ICC around 0.05 has been widely used. Assuming a completion proportion of 32%, the same number of sites would also be required to detect an increase from 32 to 48%, assuming the same power and ICC. Therefore, after accounting for one site per arm dropping out, a total of 38 sites (19 per arm) would be required. After accounting for six (20%) patient participants dropping out, 36 patient participants would be required in each site.

### Recruitment

At time of writing (October 2023), site recruitment is underway. The trial is being promoted in PR services across England and Wales through emails, phone calls, presentations at regional PR-network meetings, meetings with local Clinical Research Networks and conferences. Services interested in becoming a trial site are asked to complete an expression of interest form. PR staff in eligible sites are invited to an information meeting. The expression of interest form and information meeting are used to ascertain if the PR service meets the site eligibility criteria. The IMPROVE trial manager liaises with the local NHS research and development department in relation to assessment of capacity and capability and to get a site agreement in place. A site initiation visit will be held then. The site will be randomised then. All sites will nominate a site Principal Investigator to take overall responsibility for the leadership and conduct of the research at their site.

In sites randomised to the intervention PR staff nominated to undertake the PR-staff training will be consented as trial participants by IMPROVE research staff. Eligible PR buddies will be recruited by the trained PR staff, who will write to COPD patients who have completed PR in their service within the last year. All interested respondents will be screened for eligibility in a phone call. Those considered eligible will then be invited to interview. All those who successfully complete the interview will be invited to attend the PR-buddy training at that site. Consent will be obtained from PR buddies at the first training day.

Patient participants referred for PR will be informed about the trial at the time they are offered a pre-PR assessment appointment. Recruitment and consent of patient participants will be completed by local NHS research delivery staff or local Clinical Research Network staff who are blinded to the outcome of randomisation for the PR service.

### Assignment of interventions: allocation

During the internal pilot, block randomisation will be used. It will be applied to 8 internal pilot sites and will be carried out by the King’s Clinical Trials Unit (KCTU) using specifically designed software. Randomisation data will be held by the KCTU on a King’s College London (KCL) server.

Minimisation randomisation will take place in the main trial also at the level of research (PR) sites. It will be carried out online by the KCTU, also using specifically designed software. Randomisation data will be held by KCTU on a KCL server. Factors in minimisation will be as follows: the workplace setting of the PR service (whether it is a stand-alone PR service or integrated within a respiratory service team or community team); site situation in an urban or rural setting; and delivery of the service in cohorts or as a rolling programme (in cohorts all patients start PR at the same time, in rolling programmes patients can join the programme at any time).

The allocation sequence will be generated by KCTU. IMPROVE research staff will enter the details of the participating sites into the online minimisation randomisation system. The site will then be allocated to either the intervention or control arm of the trial. Back-up telephone access will be available where randomisation cannot be done online. A validated password website using a secure server will ensure concealment.

### Assignment of interventions: blinding

PR staff and PR buddies will know to which arm of the intervention their PR service has been allocated. Patient participants will be blinded until they consent to participate, to ensure their decision to participate is not influenced by whether they will be offered the support of a PR buddy. Staff taking consent from patient participants will not be based in the PR service or the IMPROVE team. These staff will be blinded to the outcome of randomisation until the end of patient-participant recruitment in that site.

Blinding is not related to treatment allocation, but it is important to patient-participant consent. There is no situation in which unblinding would be required for patient safety. Any instances of unblinding of the local NHS research delivery staff or Clinical Research Network taking patient-participant consent will be recorded.

### Data collection and management

Following issue of the greenlight for a site to open the site principal investigator will be asked to provide baseline data for:

1. Number of referrals in the previous 3 months
2. Proportion of patients invited to PR assessment in the previous 3 months who attended the pre-PR assessment.
3. Proportion of patients who attended PR assessment between 4 and 2 months before this ‘site baseline assessment’ and who then completed PR—that is attended 75% of sessions.

Sites will be provided with logs for the recording of uptake and completion rates of PR by trial patient participants. These logs will include the outcomes of the assessments of exercise capacity at the pre-PR assessment and end of PR assessment (if attended).

Assessment of the delivery and receipt of the PR-staff and PR-buddy training will be based on analysis of recordings of training, checklists of elements of training delivered and training reviews with staff and PR buddies as described above. PR buddies will be lent mobile phones on which to record all interactions with patient participants to allow for assessment of the fidelity of the intervention. They will also be asked to complete logs of contacts with patients they are supporting.

Questionnaires at baseline, 3 and 6 months will provide data for quality of life assessment using the Chronic Obstructive Pulmonary Disease Assessment Test and the Modified Medical Research Council breathlessness questionnaire [36, 38]; anxiety and depression using the Hospital Anxiety and Depression Scale [37]; and a bespoke health resource use evaluation.

### Plans to promote participant retention and complete follow-up

PR-staff retention will be promoted at each site by having a designated link researcher from the IMPROVE team. These researchers will attend 2 out of the 3 days of PR-buddy training to assist with completion of trial-related paperwork, such as consenting PR buddies and completion of volunteer agreements. The link researcher will visit intervention sites monthly to collect recorded phone data and to link with the PR team regarding any other research management issues.

PR buddies will attend monthly meetings at which they will be offered ongoing support from PR staff. The support will include checking on interactions with patient participants, identifying any patient participants who the PR buddy is having difficulty supporting and providing remedies, sharing experience and solutions to common problems. PR buddies who withdraw while they are supporting patients will be asked to stay in contact with these patients until they end of their PR. If they are not able to do this, then the patients will be reassigned to another PR buddy by PR staff. Patient participants who wish to withdraw will be invited to complete the follow-up data collection questionnaires before leaving the trial.

Any PR staff or PR buddies who choose not to or are unable to continue until the end of the trial will be asked to participate in a qualitative interview as part of the process evaluation. Data that are collected before patient participants withdraw will be retained for analysis.

### Data management

Quantitative data will be stored electronically on a secure bespoke trial database provided by King’s Clinical Trials Unit (KCTU). Paper data will be kept in locked filing cabinets in secure rooms accessed with staff passes at either King’s College London (KCL), the University of Leeds or in the site file at PR sites. Participant contact details will be stored in a locked filing cabinet, or on a secure university computer server.

All mobile phones lent to PR buddies will be passcode protected. IMPROVE research staff visiting sites will use encrypted laptop computers to download recordings from phones lent to the PR buddies which will be kept in a locked filing cabinet when on the university sites. Audio-recorded data will be uploaded to access-restricted servers at KCL and then deleted from the phones.

Data from PR services, such as PR attendance, will be transferred using secure NHS email.

A 10% sample of data entered into the electronic database will be checked every 3 months against source data. Data management will be audited every 12 months.

Personal information held by the research team will include necessary information for telephone and mail contact including address and telephone number. Demographic and outcome data will be anonymized before entry into a database. A participant identification key will be held in encrypted files separate from the database. All publicly available data will have been anonymized.

No laboratory samples will be used in this trial.

### Statistical methods for primary and secondary outcomes

Except for enrolled clusters/patients who are found to be ineligible or who withdraw consent at baseline visit and so never receive any treatment, and for whom no data are available, main analyses will be on an intention to treat basis to reflect the randomisation process.

Chi square test adjusting for cluster effect will be used to compare the rates of completion of PR in the two trial arms. Further, a mixed effect logistic model will be used in a sensitivity analysis to adjust for potential confounders, including age, gender and site size. In addition, adjustment for cluster-level variables will be applied for selected variables including site (cluster) size.

The estimates for primary outcome will be presented as odds ratios with 95% confidence intervals for the effect of intervention. Statistical significance will be determined at 5% level using a two-sided test throughout.

A generalised linear mixed model with linear link function will be used to analyse secondary continuous outcome measures. Working correlation matrices will be unstructured, which is not unduly restrictive given that measurements are only taken at three time points. The sandwich estimator of covariance matrix will be used to obtain appropriate (consistent) estimates of precision. Analysis of secondary endpoints will be done at the individual level.

The study has no interim analysis planned.

### Methods for additional analyses

Subgroup analyses of the principal outcome measure, completion of pulmonary rehabilitation, are planned to assess the impact of the following: the number of pulmonary rehabilitation sessions attended by patient participants; interval between offer of appointment for pre-PR assessment and first PR class; type of pulmonary rehabilitation programme (rolling or cohort).

Multiple imputation methods will be used, incorporating any available information from the baseline assessment and explanatory variables to impute missing primary or secondary outcome measures. Sensitivity analyses to assess the robustness of the results to various assumptions regarding missing data, or participants lost to follow-up, will be conducted.

### Health economic analysis

We will conduct within-trial cost-effectiveness and cost-utility analyses for the 6-month period after patient recruitment, which will be extended to 1, 3 and 5 years by a decision model if the intervention is effective at increasing PR completion. These economic evaluations will adopt the viewpoints of NHS and personal social services as well as that of society and be developed based on guidance for good practice, and decision support for NICE [40, 41].

Costs will encompass resources required to set up the intervention (e.g. training and recruitment of PR staff and PR buddies) and intervention delivery (e.g. ongoing meetings / support of PR staff and PR buddies, travel by PR buddies). Patient-level costs collected will cover meetings attended and resource use consequences for primary and secondary care, and personal social services, as well as out-of-pocket expenses (e.g. travel) borne by patients. Provider costs will be valued using national unit prices where possible to facilitate generalisability followed by published literature or if unavailable, local costs [42, 43]. Patient time will be valued using average income by age [44].

Outcomes for economic evaluation will be measured in natural units for cost-effectiveness analysis (e.g. cost per % change in completion rate, cost per % change in uptake rate). Quality-adjusted life years (QALYs) for cost-utility analysis will be measured with the EQ-5D-5L and calculated using the under the curve method with utility values taken from the UK social tariff [45, 46].

The analyses will present the incremental cost-effectiveness ratios, cost-effectiveness acceptability curves and incremental net benefit, based on willingness-to-pay thresholds of British pound sterling (GBP) 20,000–30,000 per QALY gained. Deterministic and probabilistic sensitivity analyses will examine whether the findings are sensitive to alternative approaches in handling missing data, or patient subgroups based on their completion of PR, and to examine the uncertainty in the assumptions on disease progression or service use beyond trial period and the range of the input data.

### Plans to give access to the full protocol, participant-level data and statistical code

The trial is registered with ISRCTN—12,658,458. The protocol is available. The current version of the protocol is 1.6, dated 12/5/2023. Data on rates, manner and duration of contacts between PR buddies and patient participants, details of rates of take-up and completion of PR, and the health economic data will be available after the end of the trial. KCL operates a research data repository, King’s Open Research Data System (KORDS), based on the Figshare® data repository system. It provides a self-deposit way to upload data, providing long-term secure storage and access to datasets at project-end. Depositing meets the policy requirements of funders for data retention and sharing, and the requirements of many publishers for access to datasets supporting publications. We will follow institutional guidelines for access to the data and will be informed by Medical Research Council policy on data sharing. Processes for the sharing of data will be detailed in the formal Data Management Plan.

### Oversight and monitoring

#### Composition of the coordinating centre and trial steering committee

The coordinating centre in London includes the Chief Investigator, Trial Manager, Health Psychologist Research Assistant and administrator, who provide day-to-day support for the trial. There is also a smaller research team based in Leeds with a Senior Research Fellow and Research Assistant, who provide day-to-day support for the trial. All of these core researchers meet weekly for operational meetings.

The Trial Management Group (TMG) consists of the Chief Investigator, Co-Investigators (including a patient representative), Trial Manager, Senior Research Fellow, Health Psychologist, Statistician, Health Economist and Research Assistants and trial administrator. The TMG meets 6–8 times per year to guide the management of the trial, support the achievement of milestones, oversee the delivery of key objectives, review the risk monitoring strategy, and identify solutions to any problems.

An independent trial steering committee (ITSC) comprises 4 independent members and the Chief Investigator. Their role is to provide overall supervision and ensure that the trial is conducted in accordance with the guidelines for Good Clinical Practice, Research Governance Framework for Health and Social Care and all other relevant regulations and local policies. The ITSC is responsible for advising the funder, the National Institute of Health and Care Research, on the progress of the research. The ITSC will meet to review progress and make a recommendation on the progression of the trial after approximately 18 months from the start of the trial. The ITSC will also meet with members of the research team to review and comment on the analysis of the trial principal outcome measure.

#### Composition of the data monitoring committee, its role and reporting structure

An independent data monitoring committee was not established because the trial is low risk and the analysis of the primary outcome will be a relatively simple comparison between the intervention and usual care clusters.

### Adverse event reporting and harms

All adverse events will be recorded on case report forms at the site in question. A protocol for the management of adverse events is provided to all sites. Serious adverse events (SAEs) will be reported to the trial manager by the site as soon as they are aware of them. SAEs will be reported to the sponsor within 7 days and to the main Research Ethics Committee within 15 days. A safety form for each SAE will be signed by the Chief Investigator.

Hospitalisation for a pre-existing condition, including elective procedures planned prior to study entry, which has not worsened, does not constitute an SAE. Standard supportive care for COPD does not constitute an SAE.

#### Frequency and plans for auditing trial conduct

Access to trial data for monitoring, audits and inspections will be given to authorised personnel. Trial processes may be monitored or audited according to the protocol, sponsor’s standard operating procedures, Good Clinical Practice and the application of regulatory requirements.

#### Plans for communicating important protocol amendments to relevant parties

All protocol amendments will be submitted to the sponsor for approval prior to submission to the Health Research Authority and/or Research Ethics Committee, where appropriate. The research team will inform PR sites of any amendments affecting the delivery of the trial or outcome data. Copies of the amended protocol will be sent to the ISRCTN.

### Dissemination plans

The findings of the research will be widely disseminated to all relevant stakeholders, including patients, PR staff, NHS managers, clinical commissioners, and the patient and public involvement group. The trial results will be submitted for publication in peer-reviewed journals. Findings will be presented at national and international conferences targeting both clinicians and academics. Findings will also be presented at events organised by special interest groups, seminars organised by the NHS, and events organised by patient groups. Results will also be disseminated via online platforms including the trial website.

## Discussion

The IMPROVE trial will use a hybrid effectiveness-implementation design with mixed methods, nested process, and economic evaluations [47]. This trial has the potential to improve rates of uptake and completion of PR, which is the most effective treatment known to improve symptoms and quality of life for people with COPD.

The IMPROVE trial will use a train-the-trainer model, whereby PR staff will be trained by the researchers to subsequently train volunteer PR buddies (specially trained lay health workers). Although volunteers of this kind have been used in other healthcare settings, this will be the first time (apart from the trial feasibility study) that volunteer lay health workers are used in pulmonary rehabilitation for COPD. If successful, this intervention will have the potential to be rolled out across the NHS.

The trial includes a nested process evaluation to inform the implementation of the intervention if the trial yields positive findings, or to inform the interpretation of findings if otherwise. The economic evaluation will be the first of a trial using PR conducted in the UK since 2001 [48].

The NHS Long Term Plan aims to increase access to PR over the next 10 years [49]. As services expand to meet this target, the IMPROVE intervention has the potential to help optimise the benefits of PR for patients with COPD by increasing the number of patients who are successfully completing the programme. This, in turn, should lead to benefits on quality of life, exercise tolerance and more effective self-management of COPD. The intervention has been designed so that it can be delivered feasibly by volunteers familiar with PR and who are trained and supported by PR staff. If the trial shows positive results, the intervention could be implemented by PR services throughout the NHS. The PR-buddy model also has the potential to be used in other settings including cardiac rehabilitation and rehabilitation in long COVID services.

### Trial status

Current protocol is version 1.8 dated 26th October 2023. At the time of submission, more than half of sites had been recruited and a small number of patients. Patient-participant recruitment started on 13th July 2023. Participant recruitment is scheduled to be completed on 30 September 2024.

### Supplementary Information

**Supplementary Material 1.**

## Data Availability

Data will be available after the end of the trial. King’s College London operates a research data repository, King’s Open Research Data System (KORDS), based on the Figshare® data repository system.

## Abbreviations

IMPROVE: Improving Completion of Pulmonary Rehabilitation in COPD with PR buddies
PR: Pulmonary rehabilitation
NHS: National Health Service
COPD: Chronic Obstructive Pulmonary Disease
NICE: The National Institute for Health and Care Excellence
R&D: Research and Development
DBS: Disclosure and Barring Service
CRN: Clinical Research Network
COM-B model: Capability, opportunity, and motivation behaviour change model
CRFs: Case Report Forms
ICC: Intraclass correlation coefficient
KCTU: King’s Clinical Trials Unit
KCL: King’s College London
QALYs: Quality-adjusted life years
KORDS: King’s Open Research Data System
TMG: Trial Management Group
ITSC: Independent trial steering committee
SAE: Serious adverse events
